# Vehicular Traffic–Related Polycyclic Aromatic Hydrocarbon Exposure and Breast Cancer Incidence: The Long Island Breast Cancer Study Project (LIBCSP)

**DOI:** 10.1289/ehp.1307736

**Published:** 2015-05-22

**Authors:** Irina Mordukhovich, Jan Beyea, Amy H. Herring, Maureen Hatch, Steven D. Stellman, Susan L. Teitelbaum, David B. Richardson, Robert C. Millikan, Lawrence S. Engel, Sumitra Shantakumar, Susan E. Steck, Alfred I. Neugut, Pavel Rossner, Regina M. Santella, Marilie D. Gammon

**Affiliations:** 1 Department of Epidemiology, University of North Carolina, Chapel Hill, Chapel Hill, North Carolina, USA; 2 Consulting in the Public Interest, Lambertville, New Jersey, USA; 3 Department of Biostatistics, and; 4 Carolina Population Center, University of North Carolina, Chapel Hill, Chapel Hill, North Carolina, USA; 5 Division of Cancer Epidemiology and Genetics, National Cancer Institute, National Institutes of Health, Department of Health and Human Services, Bethesda, Maryland, USA; 6 Department of Epidemiology, Mailman School of Public Health, Columbia University, New York, New York, USA; 7 Department of Preventive Medicine, Mount Sinai School of Medicine, New York, New York, USA; 8 GlaxoSmithKline Inc., Singapore; 9 Department of Epidemiology and Biostatistics, Arnold School of Public Health, University of South Carolina, Columbia, South Carolina, USA; 10 Department of Medicine, and; 11 Department of Environmental Health Sciences, Columbia University, New York, New York, USA; 12 Laboratory of Genetic Ecotoxicology, Institute of Experimental Medicine AS CR, Prague, Czech Republic

## Abstract

**Background:**

Polycyclic aromatic hydrocarbons (PAHs) are widespread environmental pollutants, known human lung carcinogens, and potent mammary carcinogens in laboratory animals. However, the association between PAHs and breast cancer in women is unclear. Vehicular traffic is a major ambient source of PAH exposure.

**Objectives:**

Our study aim was to evaluate the association between residential exposure to vehicular traffic and breast cancer incidence.

**Methods:**

Residential histories of 1,508 participants with breast cancer (case participants) and 1,556 particpants with no breast cancer (control participants) were assessed in a population-based investigation conducted in 1996–1997. Traffic exposure estimates of benzo[*a*]pyrene (B[*a*]P), as a proxy for traffic-related PAHs, for the years 1960–1995 were reconstructed using a model previously shown to generate estimates consistent with measured soil PAHs, PAH–DNA adducts, and CO readings. Associations between vehicular traffic exposure estimates and breast cancer incidence were evaluated using unconditional logistic regression.

**Results:**

The odds ratio (95% CI) was modestly elevated by 1.44 (0.78, 2.68) for the association between breast cancer and long-term 1960–1990 vehicular traffic estimates in the top 5%, compared with below the median. The association with recent 1995 traffic exposure was elevated by 1.14 (0.80, 1.64) for the top 5%, compared with below the median, which was stronger among women with low fruit/vegetable intake [1.46 (0.89, 2.40)], but not among those with high fruit/vegetable intake [0.92 (0.53, 1.60)]. Among the subset of women with information regarding traffic exposure and tumor hormone receptor subtype, the traffic–breast cancer association was higher for those with estrogen/progesterone-negative tumors [1.67 (0.91, 3.05) relative to control participants], but lower among all other tumor subtypes [0.80 (0.50, 1.27) compared with control participants].

**Conclusions:**

In our population-based study, we observed positive associations between vehicular traffic-related B[*a*]P exposure and breast cancer incidence among women with comparatively high long-term traffic B[*a*]P exposures, although effect estimates were imprecise.

**Citation:**

Mordukhovich I, Beyea J, Herring AH, Hatch M, Stellman SD, Teitelbaum SL, Richardson DB, Millikan RC, Engel LS, Shantakumar S, Steck SE, Neugut AI, Rossner P Jr., Santella RM, Gammon MD. 2016. Vehicular traffic–related polycyclic aromatic hydrocarbon exposure and breast cancer incidence: the Long Island Breast Cancer Study Project (LIBCSP). Environ Health Perspect 124:30–38; http://dx.doi.org/10.1289/ehp.1307736

## Introduction

Breast cancer is the most common malignancy among women in the United States [American Cancer Society (ACS) 2011]. Polycyclic aromatic hydrocarbons (PAHs) are ubiquitous pollutants formed from incomplete combustion (Boström et al. 2002). PAHs are genotoxic prooxidants, confirmed human lung carcinogens, and potent mammary carcinogens in laboratory animals [International Agency for Research on Cancer (IARC) 2010]. However, the association between PAHs and breast cancer in women is unclear (Gammon and Santella 2008). Previous population studies have reported associations between PAH-related exposures and breast cancer. For example, PAHs bind to DNA, including in breast tissue, and the resulting DNA adducts have been associated with breast cancer in epidemiological studies, although the research is scant (Gammon et al. 2002b; Rundle et al. 2000). PAH–DNA adducts reflect short-term exposures (Gammon et al. 2002b), whereas breast cancer is thought to develop over many years. Thus, it is of interest to evaluate longer-term PAH exposures in relation to breast cancer risk.

Vehicular traffic is a major ambient source of PAH exposure, especially near urban areas (Fromme et al. 2004). To our knowledge, all previous studies on traffic or air pollution exposure and breast cancer have reported some positive associations between breast cancer and at least one air pollution exposure surrogate (Bonner et al. 2005; Crouse et al. 2010; Lewis-Michl et al. 1996; Nie et al. 2007; Raaschou-Nielsen et al. 2011). In some cases, the effect estimates were close to the null [for example, 1.16 (0.89, 1.51); Raaschou-Nielsen et al. 2011]. The exposure assessment methods in these previous reports varied. Some investigations relied on simple traffic density data or sparse monitors, evaluated relatively brief periods of exposure, or focused on nitrogen oxides rather than carcinogenic particulate pollution. Further progress requires sophisticated modeling to reconstruct long-term cumulative exposures to ambient PAHs.

Breast cancer risk factor profiles differ by menopausal status and tumor subtype (ACS 2011; Chen and Colditz 2007), and fruits and vegetables may modify the carcinogenic effects of PAHs via antioxidant and other chemopreventive properties (Hecht 2000; Jin et al. 2006). However, whether the association between ambient PAHs and breast cancer varies by fruit/vegetable intake, tumor characteristics, or menopausal status is not well understood.

Our population-based study aimed to estimate the association between breast cancer incidence and vehicular traffic, overall and within subgroups of women classified according to fruit/vegetable intake, menopausal status, and tumor subtype. For the study reported here, we used long-term, individualized residential traffic benzo[*a*]pyrene (B[*a*]P) exposure estimates (as a proxy for exposure to particulate traffic PAHs), which were reconstructed using a historical, geographic exposure model that was consistent with a varied set of environmental measurements (Beyea et al. 2006). It is important to help clarify the association between traffic PAHs and breast cancer given the high incidence of breast cancer and broad exposure to traffic pollution worldwide.

## Materials and Methods

We used resources from the case–control component of the Long Island Breast Cancer Study Project (LIBCSP), a population-based investigation conducted among women residing in Nassau and Suffolk counties in Long Island, New York (Gammon et al. 2002a). All participating institutions provided institutional review board approval for this study, and all participants gave their written informed consent prior to study enrollment.

*Study population*. Eligible case participants were diagnosed with a first primary invasive or *in situ* breast cancer between August 1996 and July 1997 and were identified via rapid case ascertainment through contact with local pathology departments (Gammon et al. 2002a). Eligible control participants were women with no history of breast cancer and were identified using random digit dialing (Waksberg 1978) for women < 65 years old and Health Care Finance Administration records for women ≥ 65 years old. Controls were frequency matched based on the expected 5-year age distribution among the case participants. Case–control sample sizes taking into account LIBCSP subject selection procedures, participation rates, and vehicular traffic exposure data availability are presented in the Supplemental Material, Table S1.

The respondents included 1,508 case participants and 1,556 control participants (82.1% and 62.7% of eligible participants, respectively), who ranged between 20 and 98 years of age and were mostly postmenopausal (67.4%) and white (92.8%); the racial distribution reflects that of the study counties at the time of data collection (Gammon et al. 2002a). More than 50% of the study participants reported a household income ≥ $50,000 in the year prior to the study interview (Gammon et al. 2002a). Among women with traffic exposure information in the year 1995, 203 presented with *in situ* breast cancer and 1,071 presented with invasive breast cancer.

In previous LIBCSP reports, we found that breast cancer incidence was associated with early age at menarche, few or no births, and little or no breastfeeding (Gammon et al. 2002a; Shantakumar et al. 2007); increased body size (Eng et al. 2005); little or no physical activity (McCullough et al. 2012); low fruit/vegetable intake (Gaudet et al. 2004); low flavonoid intake (Fink et al. 2007); increased blood levels of PAH–DNA adducts (Gammon et al. 2002b); long-term residential environmental tobacco smoke exposure (Gammon et al. 2004); and increased grilled/smoked food intake (Steck et al. 2007).

*Study questionnaire*. Trained interviewers administered an at-home, 2-hr structured questionnaire within a few months of diagnosis (case participants) or study recruitment (control participants). Detailed questionnaire information included residential history, household income, educational attainment, race, religion, reproductive and medical histories, smoking, exogenous hormone use, and body size (Gammon et al. 2002a). A validated Block food frequency questionnaire (Block et al. 1986, 1990) was self-completed by 98% of case and control participants and assessed typical dietary intake during the year prior to the interview, including intake of fruits, fruit juices, and vegetables (Gaudet et al. 2004).

*Tumor Subtypes*. Medical records were abstracted for 1,402 case participants to ascertain tumor estrogen and progesterone receptor (ER/PR) status subtypes (Gammon et al. 2002a; Yang et al. 2011; Yasui and Potter 1999). Mutations in exons 5–8 of the tumor p53 gene (*TP53*) were determined using archived, paraffin-embedded tissue (*n* = 859) (Rossner et al. 2009). Briefly, the extracted tumor DNA was amplified using polymerase chain reaction (PCR), screened via the Surveyor Mutation Detection Kit (Transgenomic, Omaha, NE, USA), and possible mutations were confirmed with an ABI 3100 capillary sequencer (Applied Biosystems Inc, Foster City, CA, USA).

*Traffic B[*a*]P exposure assessment*. Interviewers collected information regarding participants’ lifetime residential histories in Nassau and Suffolk counties only. Addresses at which a woman resided for at least 1 year were recorded and geocoded using BLR software (now part of Geographic Data Technology Inc., Lebanon, NH). Digital street maps were based on Census “Tiger” maps (http://www.census.gov/geo/maps-data/data/tiger.html), which provide residence numbers at the start and end of a straight line street segment. The locations of intervening residence numbers were obtained by linear interpolation. The percentage of addresses that geocoded successfully ranged between 60% in the 1960s and 85% in 1995 (Beyea et al. 2005), and 87% of participants provided at least one address that geocoded to the street level.

The traffic B[*a*]P exposure model (Beyea et al. 2005, 2006, 2013) used a road network comprising approximately 500,000 street segments in the New York metropolitan area, which includes Nassau and Suffolk counties. Emissions for each street segment were calculated as the product of hourly roadway-specific traffic counts and average U.S. tailpipe emissions for the years 1960, 1970, 1980, and 1990. Estimates for other years between 1960 and 1995 were derived via interpolation or extrapolation. Vehicular traffic B[*a*]P estimates included adjustment for acceleration/deceleration at intersections (Beyea et al. 2006). Emissions within 80 km of the study area were directly calculated, and the exposure model also included a background term (proportional to exposures from the 22 counties just outside of the 2-county study area) to account for more distant roads (Beyea et al. 2006). Tailpipe emission parameters were derived from road tunnel measurements throughout the United States and were checked using measurements on vehicles run on dynamometer test beds (Beyea et al. 2008). As previously reported by Beyea et al. (2006), roadway traffic counts were obtained from over 13,000 annual average daily traffic measurements recorded in state and county records. All roads for which traffic counts were collected on Long Island by New York State, New York City, and Nassau and Suffolk counties were included in the direct emission model. Traffic counts for Nassau and Suffolk counties were taken from paper maps and entered into a geographic information system (see Supplemental Figure S1). For other counties, those roads for which counts were collected by New York State were included. Additional road counts were assumed to be proportional to the counts on major roads. Then, it was possible to scale “outer county” emissions to estimate total emissions from these counties, obtaining the scale factor as part of the fit to soil PAH data in the yards of study participants.

Roadway emissions were translated into predicted residential ambient B[*a*]P concentrations, considered a surrogate for exposure to all traffic PAHs, using standard meteorological dispersion and deposition models (Beyea et al. 2006; Chock 1978; Viegele and Head 1978). Up to 100 m from a road, traffic B[*a*]P levels were estimated using highway line-source puff models (Chock 1978). Beyond 100 m, a standard Gaussian plume model with Briggs dispersion parameters (Viegele and Head 1978), which incorporated data on wind speed and direction, rain washout, photo decay, and particle deposition, was used (Beyea et al. 2006). Meteorological data were collected at Brookhaven National Laboratory (year 1993), which was used as the standard for all years between 1960 and 1995. Changing the year (1990) or location (MacArthur Airport) of meteorological data collection for a sensitivity analysis did not appreciably alter exposure estimates (Beyea et al. 2005).

*Model validation*. The exposure model was modified to predict measured residential soil and carpet PAH levels among women living at their current address for at least 15 years, PAH–DNA adduct levels in circulating mononuclear cells collected at the time of the interview, and monitoring data for carbon monoxide, a traffic-related pollutant. Details of the model validation process are described by Beyea et al. (2006). Briefly, the model predicted soil PAHs, PAH–DNA adduct levels, and carbon monoxide concentrations, but not PAH levels in carpet dust. Specifically, when model predictions were compared with soil data, no deviation from linearity was apparent, and the *R*^2^-value was 86%. The fit to quantile data for PAH–DNA adducts was similar, though not quite as strong, with an *R*^2^-value of 58%. When examining the fit of the model to hourly CO data, the *R*^2^-value was 63%, even though the *R*^2^-value for the correlation between hourly CO emissions and concentrations was only 3.3% owing to the lag between CO emissions from tailpipes and the time of reaching distant receptors. Finally, although our model predicted that carpet PAH levels would diminish across zones of urbanization, the carpet PAH levels actually peaked at the center of the “zones” (Beyea et al. 2006). The latter finding is consistent with a study showing that residential ambient PAH concentrations do not correlate with house dust PAH levels (Fromme et al. 2004).

The model was calibrated against soil and PAH–DNA adduct data through chi-squared minimization (Beyea et al. 2006). The soil-calibrated model provided the best fit to field data and was therefore used for regressions against breast cancer incidence. Emissions during engine warm-up, which were included during model building, did not contribute to predicting soil PAH levels in validation exercises and were dropped from the exposure model (Beyea et al. 2006).

*Missing exposure data*. Residential histories during 1960–1995 were incomplete for study participants who migrated to Long Island from other locations or whose recollection of prior addresses was incomplete (Beyea et al. 2013). We estimated emissions during these time periods by imputing values for the dispersion or transfer of emissions from the entire road network to exposure at individual residences, referred to as the *D*-value. *D*-values tend to be roughly constant over historical time, changing only as the relative traffic pattern changes in different parts of the modeling region.

For time spent outside of the study area, we used a random variable with a lognormal distribution whose mean was the average estimated traffic B[*a*]P concentration on Long Island during the relevant period, based on 5-year intervals. We used multiple imputation (MI) to derive the variance of the random *D*-value, which was set equal to the variance of *D*-values for women in the study area during the same 5-year interval used to compute the mean (Beyea et al. 2013; Rubin 1996).

When the specific location of a residence within the Long Island study area was not known, the *D*-value was imputed by interpolating between values at residences before and after the missing value (Beyea et al. 2013), as suggested by Raaschou-Nielsen et al. (2011). To obtain values that differed between imputed data sets, we multiplied the interpolated *D*-value by a random number with a log normal distribution whose variance was taken from a lognormal fit to the two *D*-values used for interpolation. If the missing *D*-value was the first or last *D*-value for a study participant, extrapolation was used. For each of 30 imputed data sets, distinct values were randomly drawn from the derived lognormal distributions used to pick imputed *D*-values.

*Statistical methods*. We evaluated cumulative vehicular traffic B[*a*]P exposures for the year 1995 (the year prior to case ascertainment) and for the period 1960–1990. For 1995 exposures, we conducted a complete case analysis (CCA), in which all participants had complete, unimputed exposure information. For 1960–1990 exposures, we restricted analyses to women with ≤ 20% of their total cumulative exposure imputed (Beyea et al. 2013). We also conducted a sensitivity analysis restricting allowed imputation percentage by exposure duration rather than percentage of cumulative exposure level, as well as conducting a CCA for associations with 1960–1990 exposure. We selected a range of exposure durations in order to maximize study power, which was higher for recent exposures, in addition to benefiting from long-term cumulative exposure information. We also removed the contribution of intersections from the exposure model and imputed missing exposures according to census place (i.e., city, town, or village) (Beyea et al. 2013). Emissions at intersections contribute strongly to total traffic PAH exposure because of the increased acceleration/deceleration that takes place in these locations (Beyea et al. 2006).

We calculated descriptive statistics for the exposure variables, overall and by case–control status, and evaluated Spearman and Pearson correlations (Rothman et al. 2012) between selected exposure variables. Pearson correlation is more sensitive to outliers and the upper tails of a distribution than Spearman correlation. “Outlier” exposure estimates in our study reflected truly increased exposures for these individuals owing to proximity to heavily trafficked intersections (Beyea et al. 2006, 2013). Thus, Pearson coefficients may be informative when considering women with the highest exposure levels, but Spearman coefficients are likely to be more representative of the overall ranking for most participants.

Exposure levels were normalized to the average B[*a*]P exposure in 1995 and expressed as relative units (Beyea et al. 2013) in order to avoid interpreting results as stemming from specific B[*a*]P exposure levels; rather, B[*a*]P is to be considered a proxy for all traffic PAHs.

Statistical analyses were performed using SAS version 9.3 (SAS Institute Inc., Cary, NC).

Prior to undertaking regression analyses, we used age-adjusted cubic smoothing splines with knots at each unique exposure value (Hastie and Tibshirani 1990) to evaluate the association between traffic-related B[*a*]P exposure (during 1995 and 1960–1990) and breast cancer incidence. The spline figures for both short- and long-term exposures indicated that the association was not linear (data not shown) and were used to generate traffic B[*a*]P quantile cut points for regression models: < 50th (referent), 50th to < 75th, 75th to 95th, and ≥ 95th percentiles. We are precluded from showing these figures because of stipulations in the LIBCSP’s consent forms and IRB approval regarding presenting results for individual women or groups comprising fewer than five participants because the figures would single out women with high traffic PAH exposure levels.

We used unconditional logistic regression (Hosmer and Lemeshow 1989) to estimate odds ratios (ORs) and 95% confidence intervals (CIs) for associations between traffic B[*a*]P estimates and breast cancer incidence. When using partially imputed exposure variables, effect estimates and confidence limits were combined across 30 imputations using Rubin’s rules (Rubin 1996).

All regression models were adjusted for the case–control frequency-matching factor, 5-year age group (Gammon et al. 2002a). Other potential confounders [educational level (less than high school, high school graduate, some college, college graduate, or post-college), annual household income (< $15,000, $15,000–$19,999, $20,000–$24,999, $25,000–$34,999, $35,000–$49,999, $50,000–$69,999, $70,000–$89,999, or ≥ $90,000), race (white, black, or other), religion (none, Protestant, Catholic, Jewish, or other), parity (continuous), age at first birth (continuous), body mass index (kilograms per meter squared, continuous) duration of oral contraceptive use (continuous), lifetime average alcohol intake (never drinkers, < 15 g/day, 15–30 g/day, ≥ 30 g/day), lifetime average physical activity (in hours per week: none, low, average, and high), and duration of breastfeeding (continuous)] were identified by a thorough literature review and analysis of directed acyclic graphs (Shrier and Platt 2008) and were evaluated for model inclusion using a 10% change-in-estimate criterion relative to age-adjusted models (Rothman et al. 2012). No covariates altered age-adjusted effect estimates for the year 1995 or for the period 1960–1990 by 10% or more. We present the results as age-adjusted models as well as models adjusted for all potential confounders.

We explored effect modification by menopausal status (pre- vs. postmenopausal) and by fruit/vegetable intake in the year prior to the study interview (≤ 34 vs. ≥ 35 servings/week) for 1995 and 1960–1990 exposures using stratified analyses and likelihood ratio tests for multiplicative interaction (Rothman et al. 2012). Menopausal status was defined using data from the study questionnaire, which was collected during the case–control interview (Shantakumar et al. 2007). Specifically, menopausal status was defined according to information on participants’ last menstrual period, history of hysterectomy or bilateral oophorectomy, smoking status, and hormone replacement therapy use (Shantakumar et al. 2007). Fruit/vegetable intake was dichotomized based on previously reported associations with breast cancer incidence in the LIBCSP (Gaudet et al. 2004).

We used age-adjusted polytomous logistic regression models (Hosmer and Lemeshow 1989) to estimate associations between traffic B[*a*]P and breast cancer, with cases categorized according to tumor *TP53* mutation status and hormone receptor status subtypes and by whether tumors presented as invasive or *in situ*. Polytomous regression simultaneously generates effect estimates for several groups in relation to control participants. The ratios of the odds ratios and 95% CIs (Schlesselman 1982) for these associations were then used to formally evaluate the heterogeneity of the effect estimates across tumor types [*TP53* mutation-positive vs. *TP53* mutation-negative, *in situ* vs. invasive, ER+PR+ vs. all other subtypes (ER–/PR+, ER+/PR–, or ER–/PR–), and ER–PR– vs. all other subtypes (ER+/PR–, ER–/PR+, or ER+/PR+)]. The upper exposure quantiles (75th to < 95th and ≥ 95th percentiles) were collapsed when cell sizes comprised fewer than 10 participants.

## Results

The number of LIBCSP respondents for whom traffic B[*a*]P estimates were available (520–1,274 case participants; 566–1,334 control participants) varied according to the exposure definition and the imputed data set (some women exceeded the limit on imputation percentage in certain imputation draws), and the participants showed a wide range of exposure levels (Table 1). Mean estimated exposures were consistently higher among case particpants than control participants (Table 1). In the year 1995, mean exposures were 1.03 and 0.97 units for participants in the case and control groups, respectively. The corresponding mean levels for cumulative exposure in 1960–1990 were 227.42 and 196.71 units, where each unit corresponds to one year’s worth of average exposure in 1995.

**Table 1:** Residential vehicular traffic benzo[a]pyrene exposure estimates by case–control status, Long Island Breast Cancer Study Project, 1996–1997.

Traffic PAH exposure years^*a*^	Cases (*n* = 1,508)		Controls (*n* = 1,556)	
	*n*	Mean (IQR)	*n*	Mean (IQR)
1995 (CCA)	1,274	1.03 (0.62)	1,334	0.97 (0.55)
1960–1990 (≤ 20% MI)^*b*^	520–551	227.42 (125.31)	566–597	196.71 (122.06)
Abbreviations: CCA, complete case analysis; IQR, interquartile range; MI, multiple imputation. ^***a***^Exposure levels were normalized to the average B[*a*]P exposure in 1995 and expressed as relative units in order to avoid interpreting results as stemming from specific B[*a*]P exposure levels. Rather, B[*a*]P is to be considered a proxy for all traffic PAHs. Mean values for multiple years represent cumulative versus mean annual exposure. ^***b***^Combined over 30 imputations. Sample size varies across imputed data sets.				

Short-term (1995) and long-term [1960–1990 (20% MI)] exposure estimates were correlated (Spearman correlation coefficient: *r* = 0.76, Pearson correlation coefficient: *r* = 0.41; see Supplemental Material, Table S2).

Adjusted cubic spline figures suggested an increase in breast cancer incidence among women with the top 1% of traffic B[*a*]P exposure levels in 1995 and in 1960–1990 (data not shown). These results are based on a small number of women. Hence, we used broader quantiles (≥ 95th percentile, 75th to < 95th percentile, 50th to < 75th percentile, < 50th percentile) in regression models for 1995 and 1960–1990 in order to stabilize the results, which are presented in Table 2. Age-adjusted ORs from logistic regression models comparing participants with the top 5% of exposure levels to those with exposures below the median were 1.14 (95% CI: 0.80, 1.64) for 1995 exposure (complete case analysis), and 1.44 (95% CI: 0.78, 2.68) for cumulative exposure during 1960–1990 (among women with ≤ 20% of their total exposure based on imputed values) (Table 2). Effect estimates were similar when adjusting for the full set of potential confounders, especially for the 1960–1990 results (Table 2). Restricting participants’ imputation percentage by exposure duration rather than by percentage of cumulative exposure level did not alter results for the association between 1960–1990 traffic PAHs and incident breast cancer (data not shown). Associations for 1960–1990 (CCA) were also similar to the results for 1960–1990 with 20% MI (data not shown). After removing the contribution of intersections from the exposure model, we observed null associations between traffic PAH exposure and breast cancer risk (even among women with the highest exposures) which is consistent with a previous LIBCSP report indicating the importance of intersection emissions to total exposure (Beyea et al. 2006). However, interactions with fruit/vegetable intake were still evident (data not shown).

**Table 2:** Associations between varying time ranges of exposure to benzo[a]pyrene from residential vehicular traffic and breast cancer incidence, Long Island Breast Cancer Study Project, 1996–1997.

Traffic PAH exposure years/percentile of exposure	Age-adjusted models			Fully-adjusted models^*a*^		
	Cases	Controls	OR (95% CI)	Cases	Controls	OR (95% CI)
1995 (CCA)
< 50th	645	659	1.00 (referent)	606	620	1.00
50 to < 75th	299	353	0.87 (0.72, 1.04)	281	330	0.78 (0.63, 0.98)
75 to < 95th	260	261	1.01 (0.82, 1.23)	241	251	1.02 (0.80, 1.30)
≥ 95th	70	61	1.14 (0.80, 1.64)	67	58	1.06 (0.70, 1.60)
1960–1990 (≤ 20% MI)^*b*^
< 50th	262–287	289–320	1.00 (referent)	244–267	274–304	1.00
50 to < 75th	122–139	136–155	0.99 (0.73, 1.36)	113–130	126–142	0.97 (0.66, 1.42)
75 to < 95th	96–111	111–121	0.95 (0.68, 1.32)	88–104	104–113	0.92 (0.61, 1.39)
≥ 95th	24–29	19–21	1.44 (0.78, 2.68)	23–28	16–18	1.47 (0.70, 3.08)
Abbreviations: CCA, complete case analysis; CI, confidence interval; MI, multiple imputation; OR, odds ratio; PAH, polycyclic aromatic hydrocarbon. ^*a*^Adjusted for 5-year age group, educational level, annual household income, race, religion, parity, age at first birth, body mass index (kg/m^2^), duration of HRT use, duration of oral contraceptive use, lifetime average alcohol intake, lifetime average physical activity, and duration of breastfeeding. ^*b*^Combined over 30 imputations. Sample size varies across imputed data sets.						

The traffic B[*a*]P-breast cancer association was positive among women with low fruit/vegetable intake (Table 3), but not among women with high fruit/vegetable intake. For example, among women with low fruit/vegetable intake, we observed elevated ORs for the top 5% of 1995 exposures for this traffic PAH proxy compared with levels below the median (OR = 1.46; 95% CI: 0.89, 2.40, *p* for trend = 0.04). Among women with high fruit/vegetable intake, the corresponding OR was 0.92 (95% CI: 0.53, 1.60, *p*-interaction = 0.01). For 1960–1990 exposure, ORs for the top ≥ 75th percentile of exposure relative to exposures below the median were 1.43 (95% CI: 0.94, 2.16) among those with low fruit/vegetable intake and 0.71 (95% CI: 0.43, 1.15) among those with high fruit/vegetable intake.

**Table 3:** Associations between traffic benzo[a]pyrene exposure and breast cancer incidence within strata of fruit/vegetable intake and menopausal status, Long Island Breast Cancer Study Project, 1996–1997.

Traffic PAH exposure classification	Cases (*n*)	Controls (*n*)	Age-adjusted OR (95% CI)	Cases (*n*)	Controls (*n*)	Age-adjusted OR (95% CI)	*p* for interaction^*a*^
Fruit/vegetable intake^*b*^^,^^*c*^	Low			High		
1995 (CAA)							0.01
< 50th percentile	404	431	1.00 (referent)	228	215	1.00 (referent)
50th to < 75th percentile	193	188	1.10 (0.86, 1.41)	98	159	0.58 (0.43, 0.80)
75th to < 95th percentile	156	132	1.24 (0.94, 1.62)	99	120	0.78 (0.56, 1.08)
≥ 95th percentile	42	30	1.46 (0.89, 2.40)	28	29	0.92 (0.53, 1.60)
*p* for trend			0.04			0.14
1960–1990 (≤ 20% MI)^*d*^^,^^*e*^							0.04
< 50th percentile	169–182	174–200	1.00 (referent)	89–105	100–113	1.00 (referent)
50th to < 75th percentile	79–92	73–84	1.19 (0.80, 1.77)	35–45	59–70	0.67 (0.39, 1.13)
≥ 75th percentile	79–89	56–64	1.43 (0.94, 2.16)	42–49	66–72	0.71 (0.43, 1.15)
*p* for trend			0.09			0.12
Menopausal status^*f*^	Premenopausal			Postmenopausal		
1995 (CCA)							0.02
< 50th percentile	190	229	1.00 (referent)	439	397	1.00 (referent)
50th to < 75th percentile	101	122	0.95 (0.68, 1.32)	193	222	0.79 (0.62, 1.00)
75th to < 95th percentile	91	67	1.64 (1.13, 2.38)	166	186	0.80 (0.62, 1.02)
≥ 95th percentile	16	16	1.20 (0.58, 2.47)	51	43	1.06 (0.69, 1.63)
*p* for trend			0.04			0.21
1960–1990 (≤ 20% MI)^*d*^^,^^*e*^							0.50
< 50th percentile	49–66	56–73	1.00 (referent)	209–228	210–240	1.00 (referent)
50th to < 75th percentile	23–29	29–38	0.84 (0.42, 1.66)	95–108	101–114	1.00 (0.70, 1.42)
≥ 75th percentile	23–29	19–26	1.31 (0.63, 2.71)	97–109	109–115	0.91 (0.64, 1.29)
*p* for trend			0.60			0.60
Abbreviations: CCA, complete case analysis; CI, confidence interval; MI, multiple imputation; OR, odds ratio. ^***a***^*p*-Values for interaction were derived using likelihood ratio tests. ^***b***^ ≥ 35 versus 0–34 servings/week (Gaudet et al. 2004). ^***c***^*n* = 26 case participants and *n *= 30 control participants from the 1995 CCA were missing information for fruit/vegetable intake. ^***d***^*n* = 27 case participants and *n *= 52 control participants from the 1995 CCA were missing information for menopausal status. ^***e***^Combined over 30 imputations. Sample size varies across imputed data sets. ^***f***^The upper quantiles were combined to avoid cell sizes of < 10. The overall OR for the association between traffic exposure (≥ 75th vs. < 50th percentile) and breast cancer is 1.02 (95% CI: 0.75, 1.39).							

We observed some weak evidence that traffic B[*a*]P exposure and breast cancer incidence may be more strongly related among premenopausal women (Table 3). For example, the OR for the association between breast cancer and 1995 traffic B[*a*]P exposure (≥ 95th percentile vs. below the median) was 1.20 (95% CI: 0.58, 2.47) among premenopausal women, and 1.06 (95% CI: 0.69, 1.63) among postmenopausal women (*p*-interaction = 0.02). For 1960–1990 exposure, the ORs (≥ 75th percentile vs. below the median) were 1.31 (95% CI: 0.63, 2.71) among premenopausal women and 0.91 (95% CI: 0.64, 1.29) among postmenopausal women (*p*-interaction = 0.50).

Traffic B[*a*]P exposure in 1995 (top 5% vs. below the median) was more strongly associated with ER–/PR– breast tumors (OR = 1.67; 95% CI: 0.91, 3.05), rather than ER+/PR+, ER+/PR–, or ER–/PR+ tumors (OR = 0.80; 95% CI: 0.50, 1.27; ratio of the ORs = 2.09; 95% CI: 1.08, 4.06) (Table 4). ORs for women with exposures in the 50th to < 75th and 75th to < 95th percentile ranges were below the null value. The OR for traffic B[*a*]P in 1995 (top 5% vs. below the median) and ER+/PR+ breast tumors was 0.86 (95% CI: 0.52, 1.41), versus 1.15 (95% CI: 0.68, 1.94) for ER+/PR–, ER–/PR+, or ER–/PR– tumors (Table 4). Among case women with available traffic PAH exposure estimates, 847 out of 1,274 had information regarding their tumor hormone receptor status.

**Table 4:** Odds ratios and 95% confidence intervals for the association between traffic benzo[a]pyrene exposure (1995) and tumor hormone receptor status subtypes (n = 847 case participants), Long Island Breast Cancer Study Project, 1996–1997.

Tumor category/percentile of exposure	Cases	Controls	Age-adjusted [OR (95% CI)]	Ratio of the ORs [OR (95% CI)]
ER+ and PR+ breast cancer
< 50th	281	659	1
50th to < 75th	105	353	0.69 (0.53, 0.90)	1.02 (0.72, 1.46)
75th to < 95th	94	261	0.82 (0.62, 1.08)	1.05 (0.72, 1.52)
≥ 95th	23	61	0.86 (0.52, 1.41)	0.74 (0.40, 1.38)
ER–/PR+, ER+/PR–, or ER–/PR– breast cancer
< 50th	192	659	1
50th to < 75th	70	353	0.68 (0.50, 0.91)
75th to < 95th	61	261	0.78 (0.57, 1.08)
≥ 95th	21	61	1.15 (0.68, 1.94)
ER– and PR– breast cancer
< 50th	97	659	1
50th to < 75th	41	353	0.79 (0.54, 1.16)	1.20 (0.79, 1.82)
75th to < 95th	31	261	0.81 (0.52, 1.24)	1.00 (0.64, 1.58)
≥ 95th	15	61	1.67 (0.91, 3.05)	2.09 (1.08, 4.06)
ER+/PR–, ER–/PR+, or ER+/PR+ breast cancer
< 50th	376	659	1
50th to < 75th	134	353	0.66 (0.52, 0.83)
75th to < 95th	124	261	0.81 (0.63, 1.03)
≥ 95th	29	61	0.80 (0.50, 1.27)
Abbreviations: CI, confidence interval; ER, estrogen receptor; OR, odds ratio from polytomous logistic regression; PAH, polycyclic aromatic hydrocarbon; PR, progesterone receptor.				

This traffic PAH proxy was also more strongly related to *in situ* rather than invasive breast cancers when evaluating women within the top quantile of exposure compared with exposures below the median (Table 5). The ratios of the ORs were elevated (1.5 and higher) for both 1995 and 1960–1990 exposures. For example, for 1995 exposure, the ORs for the top quantile of exposure (vs. below the median) were 1.42 (95% CI: 0.99, 2.02) for *in situ* tumors, and 0.97 (95% CI: 0.80, 1.18) for invasive tumors (ratio of the ORs = 1.46, 95% CI: 1.02, 2.09).

**Table 5:** Associations between exposure to traffic-related polycyclic aromatic hydrocarbons (PAHs) and invasive and *in situ *breast cancer incidence,*^a^* Long Island Breast Cancer Study Project, 1996–1997.

Traffic exposure years/percentile of exposure	Cases	Controls	Age-adjusted [OR (95% CI)]	Ratio of the ORs^*b *^[OR (95% CI)]
1995 (CCA)
*In situ*
< 50th	87	659	1.0
50th to < 75th	56	353	1.20 (0.84, 1.73)	1.48 (1.03, 2.14)
≥ 75th	60	322	1.42 (0.99, 2.02)	1.46 (1.02, 2.09)
Invasive
< 50th	558	659	1.0
50th to < 75th	243	353	0.81 (0.66, 0.99)
≥ 75th	270	322	0.97 (0.80, 1.18)
75th to < 95th	209	261	0.93 (0.75, 1.15)
≥ 95th	61	61	1.14 (0.79, 1.66)
1960–1990 (≤ 20% MI)^*c*^
*In situ*
< 50th	25–35	289–320	1.0
50th to < 75th	23–27	136–155	1.71 (0.92, 3.19)	1.93 (1.03, 3.60)
≥ 75th	19–23	132–140	1.63 (0.87, 3.03)	1.64 (0.87, 3.09)
Invasive
< 50th	235–256	289–320	1.0
50th to < 75th	98–114	136–155	0.90 (0.65, 1.25)
≥ 75th	103–115	132–140	0.95 (0.69, 1.31)
75th to < 95th	80–92	111–121	0.95 (0.68, 1.32)
≥ 95th	21–25	19–21	1.35 (0.71, 2.56)
Abbreviations: CCA, complete case analysis; CI, confidence interval; MI, multiple imputation; OR, odds ratio. ^***a***^Number of women with *in situ* cancer: 1995, *n *= 203; 1960–1990, *n *= 67–85. Number of women with invasive cancer: 1995, *n *= 1,071; 1960–1990, *n *= 436–485. ^***b***^The ratio of the ORs compares quantile-specific effect estimates for *in situ* and invasive breast cancer (with invasive breast cancer treated as the referent group for the comparison). ^***c***^Combined over 30 imputations. Sample size varies across imputed data sets.				

We observed no heterogeneity of the effect estimates for tumor *TP53*-mutation status subtypes. Specifically, when compared with traffic PAH exposures below the median, effect estimates for exposures in the 50th to < 75th percentile and ≥ 75th percentile vacillated around the null value for both 1995 and 1960–1990 exposures, and the ratios of the ORs did not indicate tumor heterogeneity. For example, for 1960–1990 exposure (≥ 75th percentile vs. < 50th percentile), the observed ORs were 1.14 (95% CI: 0.52, 2.49) for tumor *TP53*-mutation positive cancer, and 1.00 (95% CI: 0.69, 1.44) for tumor *TP53*-mutation negative cancer.

## Discussion

We observed a modest positive association between high-level residential exposure to vehicular traffic B[*a*]P, as a proxy for traffic PAHs, and breast cancer incidence in this population-based study, although estimates were imprecise and varied by the exposure duration examined. The age-adjusted odds ratios for the association were 1.14 (95% CI: 0.80, 16.4) for recent 1995 traffic B[*a*]P exposure and 1.44 (95% CI: 0.78, 2.68) for long-term (1960–1990) exposure when comparing participants with exposure levels at or above the 95th percentile with participants with exposures under the median. The corresponding odds ratios for the association between recent exposure to this traffic PAH proxy and breast cancer were 1.46 (95% CI: 0.89, 2.40) among women with low fruit/vegetable intake, and 1.67 (95% CI: 0.91, 3.05) among those with hormone receptor–negative breast tumors. U.S. traffic PAH emissions are greatly reduced from the high levels of the 1960s and 1970s, but annual traffic kilometers driven is increasing (Beyea et al. 2008). Our results may have increased public health significance outside the United States, given widespread exposure to traffic pollution throughout the world and delayed introduction of vehicle pollution controls. For instance, tailpipe PAH reductions comparable to those in the United States did not occur in Europe until the late 1990s (Beyea et al. 2008).

Traffic pollution is a major ambient source of indoor and outdoor ambient PAHs (Fromme et al. 2004; Nielsen 1996), especially near cities (Boström et al. 2002). Source apportionment studies conducted in urban areas have reported that approximately 40–90% of ambient PAH levels are explained by vehicular traffic emissions, depending on various factors such as degree of urbanization or day of the week (Harrison et al. 1996; Nielsen 1996).

Previous studies have consistently reported elevated associations between residential air pollution exposure and breast cancer risk (Bonner et al. 2005; Crouse et al. 2010; Lewis-Michl et al. 1996; Nie et al. 2007; Raaschou-Nielsen et al. 2011). For example, a breast cancer study conducted in Buffalo, New York, reported elevated odds ratios when comparing the highest and lowest quartiles of overall and traffic-related particulate pollution exposure in an area with high industrial emissions [OR = 2.42; 95% CI: 0.97, 6.09 for exposure at birth, or OR = 2.57; 95% CI: 1.16, 5.69 for exposure at the time of a woman’s first birth (Bonner et al. 2005; Nie et al. 2007)]. The latter study, by Nie et al. (2007), used an adapted version of the model developed for the LIBCSP. Other investigators examined continuous traffic-related nitrogen oxide levels, reporting, for example, an elevated OR of 1.31 (95% CI: 1.00, 1.71) for each 5-ppb increase in nitrogen dioxide (Crouse et al. 2010), or an OR of 1.16 (95% CI: 0.89, 1.51) for each 100-μg/m^3^ increase in nitrogen oxides (Raaschou-Nielsen et al. 2011). Finally, a study reported an elevated association between high residential traffic density in Nassau County but not Suffolk County in Long Island, NY [pollution levels are higher in Nassau County (Lewis-Michl et al. 1996)]. The study by Lewis-Michl et al. (1996) was conducted in the same two counties as our investigation.

Some previous studies estimated exposure to air pollution, a complex mixture, using relatively sparse monitors (Bonner et al. 2005), simple traffic density data (Lewis-Michl et al. 1996), or reconstructed exposure to nitrogen oxides rather than carcinogenic particulate pollution (Crouse et al. 2010; Raaschou-Nielsen et al. 2011). Two studies compared their exposure estimates against measured, related proxies (Nie et al. 2007; Raaschou-Nielsen et al. 2011). Most studies evaluated relatively brief periods of exposure. Overall, based on an informal qualitative assessment, positive results were reported across studies despite differences in exposure assessment, exposure levels and timing, study design, and participant characteristics between study populations. When stratifying analyses by menopausal status, study results were inconsistent across investigations, suggesting that timing, magnitude of exposure, and/or pollutant mix may play important roles, although such differences could also be explained by random chance.

Strengths of our study include long-term, individualized traffic B[*a*]P exposure estimates generated from an exposure model that incorporated information regarding historical tailpipe emissions, local traffic patterns, meteorological conditions, pollutant dispersion, deposition and decay data, proximity to intersections, and background PAHs (Beyea et al. 2006). Participants also had a wide range of estimated exposures due to the gradient of urbanization across the study area (Beyea et al. 2006). In addition, the LIBCSP is a large, population-based study with detailed information regarding many covariates, which facilitates controlling for confounders, assessing effect modification, and evaluating heterogeneity within breast cancer subtypes (Gammon et al. 2002a).

We estimated stronger associations between traffic B[*a*]P exposure and breast cancer incidence among women with low fruit/vegetable intake, consistent with a previous LIBCSP report that found that associations with grilled/smoked meat intake—another PAH source—were more pronounced among participants who consumed low levels of fruits/vegetables (Steck et al. 2007). Similarly, fruit/vegetable intake was negatively associated with PAH–DNA adduct levels measured in peripheral leukocytes, both in the LIBCSP (Shantakumar et al. 2005) and in an epidemiologic study conducted in Europe (Palli et al. 2000). Components of fruits and vegetables have antioxidant and other chemopreventive properties and decrease carcinogenic effects of PAHs in animals (Hecht 2000; Jin et al. 2006). To our knowledge, ours is the first population-based breast cancer study to evaluate interactions between air pollution and fruit and vegetable intake; thus, our results require confirmation. We also observed inverse associations between traffic and breast cancer in the high fruit/vegetable intake group, which is consistent with previous research showing that PAH-related tumor induction is reduced by high antioxidant intake (Hecht et al. 2002), but may also be due to chance.

Traffic B[*a*]P–breast cancer associations appeared to be stronger among premenopausal women in our study, although the greatest difference was noted within the 75th to 95th percentile traffic exposure subgroup, rather than within the top 5%, and we also saw some unexpected negative associations between traffic exposure and breast cancer among postmenopausal women. Stronger associations with premenopausal rather than postmenopausal breast cancer were observed in previous LIBCSP reports regarding combined active/passive smoking exposure (Gammon et al. 2004) and PAH–DNA adducts (Gammon et al. 2002b). Similarly, a recent systematic review concluded that associations between environmental tobacco smoke and premenopausal, but not postmenopausal, breast cancer were “consistent with causality” (Johnson et al. 2011). We also found stronger associations with traffic B[*a*]P among women with ER–/PR– breast tumors, which are overrepresented among premenopausal women (Chen and Colditz 2007), although this finding was based on a small number of cases. Previous studies conducted in an area with high industrial emissions reported positive associations between air pollution and both premenopausal and postmenopausal breast cancer that varied according to the exposure definition used; the associations in these studies did not vary by tumor hormone-receptor subtype (Bonner et al. 2005; Nie et al. 2007).

Associations between traffic B[*a*]P and breast cancer were stronger among women with *in situ* rather than invasive breast tumors when evaluating women within the top quantile of exposure (vs. below the median). A possible explanation for these findings is that PAHs may act earlier in the carcinogenic process (Millikan et al. 1995), similar to the way in which smoking may sometimes be more strongly related to colorectal adenomas, rather than to invasive colorectal cancer (Terry and Neugut 1998). Other possible explanations are the influence of potential diagnostic bias in identifying *in situ* breast tumors, or random chance. The sample size was much smaller in the subgroup of participants with *in situ* breast cancer than in the subgroup of participants with invasive breast cancer; thus, the results among the *in situ* group are more unstable. In addition, we were unable to evaluate regressions for participants with the top 5% of exposures when evaluating *in situ* breast cancer owing to sample size constraints. Instead, we evaluated broader quantiles of exposure (< 50th, 50th to < 75th, ≥ 75th percentiles), which may have obscured some patterns of association. Associations between traffic B[*a*]P and invasive breast cancer were positive, though imprecise, among those with the top 5% of exposure levels, compared with below the median (1995: OR = 1.14, 95% CI: 0.79, 1.66; 1960–1990: OR = 1.35, 95% CI: 0.71, 2.56).

Traffic B[*a*]P was not related to tumor *TP53*-mutation status in our study, although the number of case women with *TP53*-mutation–positive tumor tissue and high traffic exposure estimates was low. This null result was consistent with an LIBCSP report evaluating active and passive smoking, grilled/smoked meat intake, and PAH–DNA adducts (Mordukhovich et al. 2010). Active, but not passive, smoking was associated with *TP53*-mutation positive breast cancer in a smaller population study (Conway et al. 2002).

Several mechanisms potentially underlie the observed association between traffic-related B[*a*]P, as a proxy for overall particulate vehicular traffic PAH exposure, and breast cancer incidence. PAHs are genotoxic, damaging DNA primarily through the formation of PAH–DNA adducts; they may also induce inflammation and oxidative stress, resulting in oxidative DNA lesions (IARC 2010). Uncorrected DNA damage can cause somatic mutations in tumor suppressor genes or proto-oncogenes, which could in turn contribute to carcinogenesis (Gammon and Santella 2008; IARC 2010).

Limitations of our study include some missing address information, which was corrected in part through multiple imputation. Our results were robust to varying the percentage of imputation allowed, increasing confidence that our imputation method does not introduce artificial results (Beyea et al. 2013). Similarly, although analyses for exposure in 1960–1990 included roughly half of the study participants and may not be representative of the full study population (Gammon et al. 2002a), only evaluating 1 recent year of exposure also carries implicit assumptions and associated biases related to missing exposures.

An additional limitation of our study is that the exposure model did not generate non-residential traffic PAH exposure estimates. A nationwide study reported that, on average, American men and women spend almost 70% of their day inside (64.97%) or just outside (2.50%) of their residences, both throughout the United States and in one large city (Chicago, IL) (Leech et al. 2002). A Canadian study found similar results among women only (Nethery et al. 2009). Our results were not adjusted for multiple comparisons, although we used a targeted approach and carefully selected factors that were likely to interact with traffic PAHs. Our results may also be influenced by potential selection bias, given that our response rates among the control participants were lower than anticipated (Gammon et al. 2002a). The hormone receptor subtype results may be subject to selection bias as well, given that only a subset of case participants (847 out of 1,274) were available for these analyses. However, responses among the control participants are comparable to those among other large population-based case–control studies conducted in the 1990s (Newman et al. 1995).

The purpose of our exposure model was to estimate vehicular traffic PAH exposures; thus, by design, we did not incorporate information on non-traffic sources of ambient PAHs, such as environmental tobacco smoke, cooking, or heating (Beyea et al. 2013). Traffic is often the largest source of ambient PAHs near urban areas (Fromme et al. 2004; Nielsen 1996) and generates smaller, more inflammatory particles than some other ambient sources (Kocbach et al. 2006).

B[*a*]P was modeled as a surrogate for all traffic PAHs. Traffic pollution contains many chemicals, including many PAHs and other confirmed carcinogens (Boström et al. 2002). It is impossible to ascribe health effects to a single traffic pollutant based on an observational study. However, B[*a*]P is one of the most carcinogenic PAHs and is representative of overall PAH exposure from vehicle exhaust (Boström et al. 2002), and particulate PAHs are among the most carcinogenic components of traffic exhaust (Boothe and Shendell 2008). Studies conducted throughout the United States indicate that historical traffic-related B[*a*]P emissions closely track emissions for all other measured traffic-related PAHs (Beyea et al. 2008). Furthermore, many types of traffic pollutants have differing distributions (Beyea et al. 2006). In our study, exposures were modeled using PAH-specific emissions data and dispersion parameters and were validated and calibrated against soil PAH levels (Beyea et al. 2006). Although studies of air pollution exposure and traffic pollution have been conducted in the United States, Canada, and Europe, where traffic emission levels are currently fairly low, future studies should examine associations between traffic exposure and breast cancer within more highly exposed populations.

## Conclusions

We observed modest positive associations between breast cancer incidence and comparatively high levels of residential traffic B[*a*]P, particularly among women with low fruit and vegetable intake and hormone receptor–negative tumors, although the effect estimates were imprecise and several exposure durations and participant subgroups were evaluated. Future studies should examine associations between breast cancer and traffic PAH exposure at different points across the life-course and in combination with other PAH sources, and should evaluate interactions between traffic PAHs and genetic variants in relevant pathways. Our results strengthen the plausibility of a relationship between vehicular traffic PAHs and breast cancer incidence and provide new data on potentially susceptible subgroups of women.

## Supplemental Material

(292 KB) PDF
