# Supplementary material for: Vehicular Traffic–Related Polycyclic Aromatic Hydrocarbon Exposure and Breast Cancer Incidence: The Long Island Breast Cancer Study Project (LIBCSP)
Source: Environ Health Perspect. 2015 May 22;124(1):30–8. doi: 10.1289/ehp.1307736 (PMC4710589; doi:10.1289/ehp.1307736)
Supplement: (292 KB) PDF [file ehp.1307736.s001.acco.pdf]

Environ Health Perspect
DOI: 10.1289/ehp.1307736

Note to Readers: *EHP* strives to ensure that all journal content is accessible to all readers. However, some figures and Supplemental Material published in *EHP* articles may not conform to 508 standards due to the complexity of the information being presented. If you need assistance accessing journal content, please contact ehp508@niehs.nih.gov. Our staff will work with you to assess and meet your accessibility needs within 3 working days.

Supplemental Material

Vehicular Traffic-Related Polycyclic Aromatic Hydrocarbon Exposure and Breast Cancer Incidence: The Long Island Breast Cancer Study Project (LIBCSP)

Irina Mordukhovich, Jan Beyea, Amy H. Herring, Maureen Hatch, Steven D. Stellman, Susan L. Teitelbaum, David B. Richardson, Robert C. Millikan, Lawrence S. Engel, Sumitra Shantakumar, Susan E. Steck, Alfred I. Neugut, Pavel Rossner Jr., Regina M. Santella, and Marilie D. Gammon

Table of Contents

Figure S1. Traffic-count measurement density in a 25-km wide section of the study area. Each symbol represents a measurement location. Long Island Breast Cancer Study Project, 1996-1997.

Table S1. Case-control sample sizes following participant recruitment procedures, participation rates, and exposure data availability, Long Island Breast Cancer Study Project, 1996-1997.

Table S2. Correlations between selected residential vehicular traffic PAH exposure variables, Long Island Breast Cancer Study Project, 1996-1997.

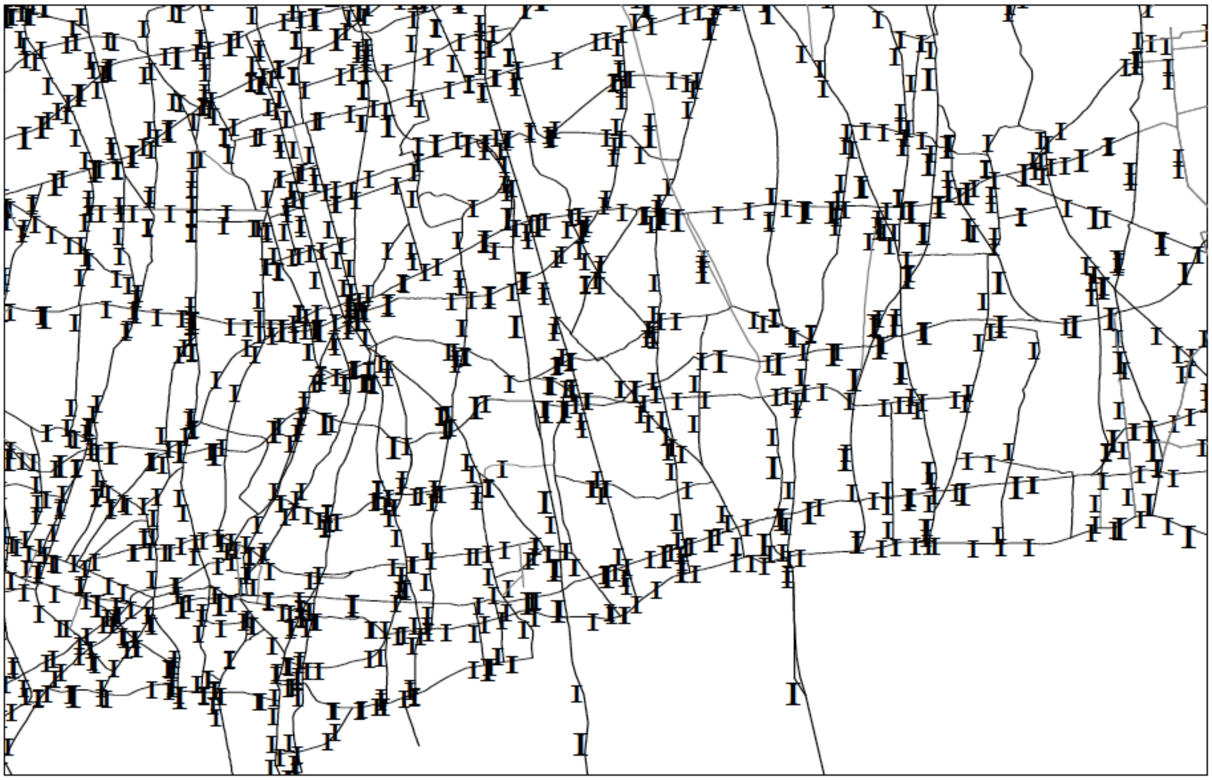

Figure S1. Traffic-count measurement density in a 25-km wide section of the study area. Each symbol represents a measurement location. Long Island Breast Cancer Study Project, 1996-1997.

Table S1. Case-control sample sizes following participant recruitment procedures, participation rates, and exposure data availability[a], Long Island Breast Cancer Study Project, 1996-1997.

Study participant status	Cases (n)		Controls (n)	
Sampled	2,271		2,714	
Eligible[b]	1,837		2,481	
Refused		217		530
Deceased		16		25
Out of area/untrackeable		25		193
Too ill or cognitively impaired		60		168
Partially completed questionnaire		10		7
Other		1		2
Completed study questionnaire	1,508		1,556	
Complete traffic PAH exposure information				
1995 (CCA)	1,274		1,334	
1980-1995 (20 % MI)[c]	961-973		1004-1016	
1980-1995 (CCA)	846		873	
1960-1990 (20 % MI)[c]	520-551		566-597	
1960-1990 (CCA)	286		273	

CCA: complete case analysis; MI: multiple imputation; PAH: polycyclic aromatic hydrocarbon

a) Adapted from Gammon et al. 2002.

b) Eligibility was determined by whether a woman was identified as having breast cancer on a hospital pathology report, whether it could be confirmed that she had a first primary breast cancer (through a medical record and/or her physician), and whether her physician consented for the LIBCSP to contact her.

c) Combined over m=30 imputations. Sample size varies across imputed data sets.

Table S2. Correlations between selected residential vehicular traffic PAH exposure variables, Long Island Breast Cancer Study Project, 1996-1997.

Traffic PAH Exposure Years	1995	1980-1995 CCA	1960-1995 CCA	1960-1990 CCA	1960-1990 20% MI[a]
**Spearman correlations**[b]					
1995	1	0.95	0.85	0.84	0.76
1980-1995 CCA		1	0.92	0.92	0.88
1960-1995 CCA			1	1.00	1.00
1960-1990, CCA				1	1.00
1960-1990, ≤20% MI[a]					1
**Pearson correlations**[b]					
1995	1	0.91	0.59	0.58	0.41
1980-1995 CCA		1	0.71	0.69	0.70
1960-1995 CCA			1	1.00	1.00
1960-1990 CCA				1	1.00
1960-1990 ≤20% MI[a]					1

CCA: complete case analysis; MI: multiple imputation; PAH: polycyclic aromatic hydrocarbon

a) Combined over m=30 imputations; sample size varies across the imputed data sets.

b) Pearson correlation is more sensitive to outliers than Spearman correlation. Outlier exposure estimates in our study reflect truly increased exposures for these individuals due to proximity to heavily trafficked intersections. Thus, Pearson coefficients may be informative when considering women with the highest exposure levels, but Spearman coefficients are probably more representative of the overall ranking for most participants.
